# Definitions of arginine availability: a critical metabolic marker of physiological processes

**DOI:** 10.3389/fendo.2026.1812528

**Published:** 2026-05-21

**Authors:** Mustafa Tosur, Farook Jahoor, Ashok Balasubramanyam

**Affiliations:** 1 Division of Diabetes and Endocrinology, Department of Pediatrics, Baylor College of Medicine, Houston, TX, United States; 2 Children’s Nutrition Research Center, Department of Pediatrics, Baylor College of Medicine, USDA/ARS, Houston, TX, United States; 3 Division of Diabetes, Endocrinology and Metabolism, Baylor College of Medicine, Houston, TX, United States

**Keywords:** arginine, arginine availability, nitric oxide, stable isotope, tracer

## Introduction

Arginine is a semi-essential amino acid whose demand may exceed supply during stress conditions including trauma, critical illness, and surgical stress (1, 2). It plays vital roles in growth, wound healing, immunity, nitric oxide (NO) synthesis and ammonia detoxification (3). It serves as a precursor for creatine, glutamate/proline, glutathione (via glutamate), and polyamines (3). Arginine is the sole substrate for NO synthesis, which is a crucial molecule for endothelial, immune, inflammatory and neural functions (4). It also stimulates insulin secretion through NO-dependent and independent mechanisms in β-cells (5, 6). These diverse roles have generated significant interest in arginine metabolism and its effects on health and disease.

## Arginine homeostasis and the concept of arginine availability

Arginine homeostasis is maintained through the balance between its catabolism and inflow from dietary intake, protein breakdown and *de novo* synthesis (3). Four major enzymes metabolize arginine: NO synthase (producing citrulline and NO), arginase (producing urea and ornithine), arginine:glycine amidotransferase, and arginine decarboxylase.

The relationship between extracellular arginine concentration and its functional effects is complicated by its complex intracellular compartmentalization and the inaccessibility of specific cellular pools for sampling. The concept of “arginine availability” has emerged to describe the amount of arginine accessible to cells and tissues for physiological processes. In the absence of a single, standardized definition of this concept, a number of surrogate estimates have been proposed to quantify arginine availability. Here, we summarize four extant definitions of arginine availability and discuss their practical relevance and relative accuracy (**Table 1**)

**Table 1:** Comparison of definitions of arginine availability.

Definition	Formula	Required measurements	Advantages	Limitations	Clinical applicability
Definition-1 (Morris et al.)^7^	Arg / (Orn + Cit)	Plasma amino acid concentrations	Accounts for arginase activity (ornithine) and renal contribution (citrulline), simple to calculate	Does not account for synthesis or metabolism; limited mechanistic depth	High – feasible in clinical and epidemiological studies
Definition-2 (Grasemann et al.)^8^	Arg / Orn or Arg / (Orn + Lys)	Plasma amino acid concentrations	Reflects competition at cationic amino acid transporters; simple to calculate	Does not account for synthesis or metabolism; limited mechanistic depth	High – practical for clinical studies
Definition-3 (Mulukutla et al.)^9^	Arginine flux − ornithine flux	Stable isotope tracer kinetics	Incorporates whole-body arginine metabolism; dynamic assessment	Assumes ornithine derives solely from arginine; ignores alternative sources and downstream metabolism, methodologically complex; limited availability	Low to moderate – limited to specialized research settings
Definition-4 (Proposed refinement)	Arginine flux − arginine oxidation (CO_2_)	Stable isotope tracer kinetics	Comprehensive; accounts for total arginine flux and irreversible loss; avoids assumptions about ornithine origin	Methodologically complex; limited availability	Low to moderate – limited to specialized research settings

Definition-1 (Morris et al.) (7): Global Arginine Availability (GAA) is defined as the ratio of plasma arginine to the sum of ornithine and citrulline, multiplied by 100. This accounts for competition of ornithine with arginine for cellular transport and its role as a marker of plasma arginase activity. Citrulline is included because it serves as both a NO synthase byproduct and an arginine substrate. Arginine synthesis from citrulline occurs in the kidneys, and plasma citrulline concentrations increase in parallel with creatinine concentrations, suggesting that impaired renal function adversely affects the conversion of citrulline to arginine. Hence, citrulline is included in the denominator to adjust for the effect of impaired renal function on arginine availability.
Morris et al. used this definition in their study of 280 patients with sickle cell disease (7). Plasma arginase activity was significantly higher in this cohort, with the highest activity observed in those with secondary pulmonary hypertension. In addition, global arginine bioavailability was strongly associated with higher mortality (Risk ratio:3.6, 95% CI:1.5-8.3, p<0.001). Notably, plasma arginase activity was correlated with hemolysis rate, linking sickle cell pathogenesis to reduced NO availability, endothelial dysfunction and pulmonary hypertension.
Definition-2 (Grasemann et al.) (8): Arginine availability is defined as the ratio of arginine to ornithine, or alternatively to the sum of ornithine and lysine, based on plasma concentrations. This formula is based on competition between arginine and ornithine/lysine for the same cationic amino acid transporter, which is a rate-limiting step in cellular arginine uptake.
Grasemann et al. (8) studied 10 patients with cystic fibrosis before and after antibiotic treatment, and compared them to healthy controls. They demonstrated increased plasma arginase levels in cystic fibrosis during pulmonary exacerbation compared to controls. In addition, arginine availability was significantly lower in cystic fibrosis before and after exacerbation, suggesting altered arginine metabolism in this condition.
Definition-3 (Mulukutla et al.) (9): Arginine availability is defined as total arginine flux minus endogenous ornithine flux, assuming ornithine derives only from arginine. This requires kinetic data derived using tracer methodology.
Our group studied arginine metabolism in adults with A-β+ ketosis-prone diabetes (KPD) using stable isotope tracer methods (9). Despite higher arginine availability in patients with KPD during euglycemic state compared to controls, arginine availability decreased more in KPD in response to hyperglycemia. While patients with KPD had lower first-phase insulin secretion to glucose stimulation in both euglycemia and hyperglycemia, intravenous exogenous arginine administration restored first-phase insulin secretion in patients with KPD to the levels similar to controls.
Definition-4 (a revised version of Definition-3.): Definition-4 represents a proposed refinement of tracer-based approaches. Arginine availability is defined as arginine flux minus its terminal oxidation to carbon dioxide (CO_2_) in breath. This revision recognizes that ornithine can be synthesized from non-arginine sources (proline and glutamate); it also requires kinetic data from tracer measurements. This definition is based on ongoing work from our group in patients with type 2 diabetes and ketosis-prone diabetes, and has not yet been formally described in the published literature.

## Discussion

Interpretation of arginine metabolism in physiological and pathological states requires consideration beyond circulating arginine concentrations, as intracellular compartmentalization limits direct inference of functional availability. The concept of arginine availability provides a framework linking measurable parameters (e.g., plasma amino acids or fluxes) to biologically relevant processes such as endothelial dysfunction and insulin secretion. Thus, selecting an appropriate definition of arginine availability is essential for accurately interpreting its role across different physiological and disease states.

Arginine availability is an inferred construct derived from measurable parameters such as plasma amino acid concentrations and metabolic fluxes. While detailed methodological considerations are beyond the scope of this opinion article, these definitions are grounded in established analytical approaches and have been applied as biomarkers in diverse pathological conditions. (5–7).

The first two definitions are practical, calculable from plasma concentration measurement, and provide a general assessment. The fourth definition is the most comprehensive and accurate, accounting for all available arginine by subtracting its oxidation (calculated from the end-product CO_2_ in breath) from its flux using stable isotope tracer methodology. Investigators may utilize any of these definitions depending on the available data (static vs. kinetic measurements) and the desired analytical depth and precision, recognizing the caveats associated with each. Definition-4 likely provides the most accurate measurement of arginine bioavailability to assess the effect of arginine on dynamic physiological processes, but its application is currently limited to specialized research settings due to methodological complexity.

In conclusion, the concept of arginine availability has emerged to better characterize the accessibility of arginine for physiological processes in the setting of complex intracellular compartmentalization. Multiple definitions have been proposed, each reflecting distinct aspects of arginine biology, including transport, metabolism, and whole-body kinetics. The appropriate definition should be selected based on the study goals, setting, and availability of kinetic data. Continued refinement and validation of these approaches may improve the interpretation of arginine metabolism and its role in health and disease.
